# Supplementary material for: Modeling glioblastoma heterogeneity as a dynamic network of cell states
Source: Mol Syst Biol. 2021 Sep 16;17(9):e10105. doi: 10.15252/msb.202010105 (PMC8444284; doi:10.15252/msb.202010105)
Supplement: Supplementary file 6 — Source Data for Figure 5 [file MSB-17-e10105-s004.zip › Figure5A_sourcedata/GSEA_3071/hallmarks_stateV.GseaPreranked.1623742546329/HALLMARK_IL2_STAT5_SIGNALING.html]

Details for gene set HALLMARK\_IL2\_STAT5\_SIGNALING[GSEA]

|  || Dataset | state43071 |
| Phenotype | NoPhenotypeAvailable |
| Upregulated in class | na\_neg |
| GeneSet | HALLMARK\_IL2\_STAT5\_SIGNALING |
| Enrichment Score (ES) | -0.49013945 |
| Normalized Enrichment Score (NES) | -2.632976 |
| Nominal p-value | 0.0 |
| FDR q-value | 0.0 |
| FWER p-Value | 0.0 |
Table: GSEA Results Summary

  

Fig 1: Enrichment plot: HALLMARK\_IL2\_STAT5\_SIGNALING      
 Profile of the Running ES Score & Positions of GeneSet Members on the Rank Ordered List

  

| PROBE | GENE SYMBOL | GENE\_TITLE | RANK IN GENE LIST | RANK METRIC SCORE | RUNNING ES | CORE ENRICHMENT || 1 | CDC6 |  |  | 328 | 1.040 | -0.0457 | No |
| 2 | RGS16 |  |  | 348 | 1.018 | -0.0326 | No |
| 3 | CCNE1 |  |  | 461 | 0.901 | -0.0392 | No |
| 4 | SYT11 |  |  | 471 | 0.889 | -0.0262 | No |
| 5 | GPX4 |  |  | 701 | 0.746 | -0.0578 | No |
| 6 | APLP1 |  |  | 792 | 0.704 | -0.0635 | No |
| 7 | ST3GAL4 |  |  | 839 | 0.684 | -0.0610 | No |
| 8 | ITGA6 |  |  | 981 | 0.627 | -0.0777 | No |
| 9 | CASP3 |  |  | 1057 | 0.605 | -0.0821 | No |
| 10 | PHTF2 |  |  | 1094 | 0.594 | -0.0792 | No |
| 11 | TNFRSF21 |  |  | 1166 | 0.573 | -0.0834 | No |
| 12 | SPRY4 |  |  | 1283 | 0.536 | -0.0968 | No |
| 13 | DCPS |  |  | 1284 | 0.536 | -0.0879 | No |
| 14 | IGF1R |  |  | 1430 | 0.497 | -0.1075 | No |
| 15 | UMPS |  |  | 1448 | 0.493 | -0.1027 | No |
| 16 | POU2F1 |  |  | 1925 | 0.400 | -0.1873 | No |
| 17 | BATF3 |  |  | 1940 | 0.398 | -0.1834 | No |
| 18 | CCND3 |  |  | 1991 | 0.390 | -0.1866 | No |
| 19 | ETV4 |  |  | 1998 | 0.389 | -0.1813 | No |
| 20 | IKZF4 |  |  | 2031 | 0.383 | -0.1811 | No |
| 21 | DENND5A |  |  | 2038 | 0.382 | -0.1760 | No |
| 22 | ECM1 |  |  | 2564 | 0.315 | -0.2715 | No |
| 23 | AHCY |  |  | 2598 | 0.311 | -0.2727 | No |
| 24 | BCL2 |  |  | 2680 | 0.302 | -0.2832 | No |
| 25 | CCND2 |  |  | 2876 | 0.282 | -0.3159 | No |
| 26 | SYNGR2 |  |  | 2933 | 0.276 | -0.3221 | No |
| 27 | NCS1 |  |  | 3150 | 0.255 | -0.3593 | No |
| 28 | RORA |  |  | 3171 | 0.253 | -0.3590 | No |
| 29 | SWAP70 |  |  | 3329 | -0.271 | -0.3846 | No |
| 30 | GLIPR2 |  |  | 3582 | -0.312 | -0.4278 | No |
| 31 | BHLHE40 |  |  | 3620 | -0.318 | -0.4297 | No |
| 32 | TNFSF10 |  |  | 3651 | -0.323 | -0.4301 | No |
| 33 | NFIL3 |  |  | 3700 | -0.331 | -0.4338 | No |
| 34 | PUS1 |  |  | 3767 | -0.343 | -0.4408 | No |
| 35 | MAFF |  |  | 3849 | -0.359 | -0.4504 | No |
| 36 | FURIN |  |  | 3861 | -0.361 | -0.4466 | No |
| 37 | NOP2 |  |  | 3928 | -0.373 | -0.4531 | No |
| 38 | GSTO1 |  |  | 4020 | -0.393 | -0.4640 | No |
| 39 | SNX9 |  |  | 4031 | -0.395 | -0.4594 | No |
| 40 | PTRH2 |  |  | 4046 | -0.399 | -0.4555 | No |
| 41 | GABARAPL1 |  |  | 4054 | -0.400 | -0.4503 | No |
| 42 | RNH1 |  |  | 4065 | -0.402 | -0.4456 | No |
| 43 | SERPINB6 |  |  | 4086 | -0.406 | -0.4427 | No |
| 44 | ITGAV |  |  | 4207 | -0.442 | -0.4584 | No |
| 45 | IFNGR1 |  |  | 4240 | -0.452 | -0.4571 | No |
| 46 | BCL2L1 |  |  | 4248 | -0.456 | -0.4509 | No |
| 47 | MYO1C |  |  | 4421 | -0.508 | -0.4755 | No |
| 48 | IFITM3 |  |  | 4424 | -0.509 | -0.4675 | No |
| 49 | SLC39A8 |  |  | 4452 | -0.521 | -0.4641 | No |
| 50 | ENPP1 |  |  | 4538 | -0.558 | -0.4712 | No |
| 51 | ARL4A |  |  | 4593 | -0.582 | -0.4719 | No |
| 52 | P4HA1 |  |  | 4689 | -0.641 | -0.4796 | Yes |
| 53 | CAPG |  |  | 4709 | -0.650 | -0.4725 | Yes |
| 54 | NCOA3 |  |  | 4720 | -0.661 | -0.4635 | Yes |
| 55 | PLAGL1 |  |  | 4723 | -0.661 | -0.4530 | Yes |
| 56 | F2RL2 |  |  | 4778 | -0.697 | -0.4518 | Yes |
| 57 | PIM1 |  |  | 4783 | -0.699 | -0.4410 | Yes |
| 58 | RHOB |  |  | 4820 | -0.731 | -0.4359 | Yes |
| 59 | LIF |  |  | 4882 | -0.789 | -0.4346 | Yes |
| 60 | CKAP4 |  |  | 4919 | -0.831 | -0.4277 | Yes |
| 61 | TGM2 |  |  | 4928 | -0.842 | -0.4154 | Yes |
| 62 | KLF6 |  |  | 4933 | -0.849 | -0.4021 | Yes |
| 63 | ITIH5 |  |  | 4934 | -0.850 | -0.3881 | Yes |
| 64 | SOCS2 |  |  | 4935 | -0.851 | -0.3741 | Yes |
| 65 | SLC2A3 |  |  | 4942 | -0.859 | -0.3610 | Yes |
| 66 | ALCAM |  |  | 4988 | -0.911 | -0.3546 | Yes |
| 67 | MYC |  |  | 5020 | -0.972 | -0.3445 | Yes |
| 68 | EMP1 |  |  | 5023 | -0.979 | -0.3288 | Yes |
| 69 | HOPX |  |  | 5091 | -1.126 | -0.3230 | Yes |
| 70 | ANXA4 |  |  | 5106 | -1.172 | -0.3064 | Yes |
| 71 | SLC1A5 |  |  | 5145 | -1.294 | -0.2923 | Yes |
| 72 | GADD45B |  |  | 5155 | -1.335 | -0.2720 | Yes |
| 73 | PLEC |  |  | 5157 | -1.349 | -0.2499 | Yes |
| 74 | XBP1 |  |  | 5165 | -1.393 | -0.2283 | Yes |
| 75 | SOCS1 |  |  | 5184 | -1.475 | -0.2074 | Yes |
| 76 | NRP1 |  |  | 5211 | -1.609 | -0.1858 | Yes |
| 77 | SCN9A |  |  | 5232 | -1.797 | -0.1600 | Yes |
| 78 | AHNAK |  |  | 5268 | -2.309 | -0.1286 | Yes |
| 79 | SPP1 |  |  | 5272 | -2.446 | -0.0888 | Yes |
| 80 | COL6A1 |  |  | 5283 | -2.747 | -0.0454 | Yes |
| 81 | PTCH1 |  |  | 5288 | -2.878 | 0.0013 | Yes |
Table: GSEA details [plain text format]

  

Fig 2: HALLMARK\_IL2\_STAT5\_SIGNALING: Random ES distribution      
 Gene set null distribution of ES for **HALLMARK\_IL2\_STAT5\_SIGNALING**
